# Epigenetic Histone Marks of Extended Meta-Polycentric Centromeres of *Lathyrus* and *Pisum* Chromosomes

**DOI:** 10.3389/fpls.2016.00234

**Published:** 2016-03-01

**Authors:** Pavel Neumann, Veit Schubert, Iva Fuková, Jasper E. Manning, Andreas Houben, Jiří Macas

**Affiliations:** ^1^Laboratory of Molecular Cytogenetics, Biology Centre of the Czech Academy of Sciences, Institute of Plant Molecular BiologyČeské Budějovice, Czech Republic; ^2^Leibniz Institute of Plant Genetics and Crop Plant Research (IPK)Gatersleben, Germany

**Keywords:** centromere structure, epigenetic modifications, histone phosphorylation, histone methylation, meta-polycentric chromosomes, holocentric

## Abstract

Species of the legume genera *Lathyrus* and *Pisum* possess chromosomes that exhibit a unique structure of their centromeric regions, which is clearly apparent during metaphase by the formation of extended primary constrictions which span up to a third of the length of the chromosome. In addition, these species express two different variants of the CenH3 protein which are co-localized in multiple domains along the poleward surface of the primary constrictions. Here, we show that the constrictions represent a distinct type of chromatin differing from the chromosome arms. In metaphase, histone phosphorylation patterns including H3S10ph, H3S28ph, and H3T3ph were observed along the entire constriction, in a way similar to holocentric chromosomes. On the other hand, distribution of phosphorylated H2AT120 was different from that previously reported from either, holocentric and monocentric chromosomes, occurring at chromatin surrounding but not overlapping CenH3 domains. Since some of these phosphorylations play a role in chromatid cohesion, it can be assumed that they facilitate correct chromosome segregation by ensuring that multiple separate CenH3 domains present on the same chromatid are oriented toward the same pole. The constrictions also displayed distinct patterns of histone methylation marks, being enriched in H3K9me2 and depleted in H3K4me3 and H3K27me2 compared to the chromosome arms. Super-resolution fluorescence microscopy revealed that although both CenH3 protein variants are present in all CenH3 domains detected on metaphase chromosomes, they are only partially co-localized while there are chromatin subdomains which are mostly made of only one CenH3 variant. Taken together, these data revealed specific features of extended primary constrictions of *Lathyrus* and *Pisum* and support the idea that they may represent an intermediate stage between monocentric and holocentric chromosomes.

## Introduction

Centromeres are defined as sites of kinetochore formation, facilitating the attachment of a spindle that drives chromosome segregation during cell division. The kinetochores of most eukaryotes are formed upon a specific type of chromatin marked by the presence of CenH3, a centromeric variant of the canonical histone H3 (Jiang et al., 2003; Henikoff and Dalal, 2005; Black and Bassett, 2008). Two types of centromere organization are generally distinguished. In monocentric chromosomes, CenH3 is located in a single region corresponding to a primary constriction on metaphase chromosomes. On holocentric chromosomes, CenH3 is deposited along almost the entire length of the chromosome, forming a lateral groove on each chromatid of mitotic chromosomes where spindle fibers attach (Cuacos et al., 2015). In terms of their underlying nucleotide sequences, the regional centromeres of monocentric chromosomes are typically enriched with satellite repeats and specific lineages of mobile elements. However, these repeats may be absent or may differ considerably between chromosomes within the species (Gong et al., 2012), thus probably not playing crucial role in centromere determination which is rather facilitated by epigenetic factors (Birchler et al., 2011; Birchler and Han, 2013). In the few plant species with holocentric chromosomes investigated so far, no specific repeats have been found to be associated with CenH3-loci in *Luzula elegans* (Heckmann et al., 2013), while CenH3 chromatin in *Rhynchospora* was shown to be enriched in one family of satellite repeats and several mobile elements (Marques et al., 2015).

In addition to the presence of CenH3 protein marking functional centromere domains, primary constrictions, and their adjacent regions, referred to as pericentromeres, display specific patterns of cell cycle-dependent post-translational histone modifications that distinguish them from the modifications found within chromosome arms. In plants, the distribution of histone H3 phosphorylated at S10 (Houben et al., 1999; Kaszás and Cande, 2000; Manzanero et al., 2000; Kurihara et al., 2006) and S28 (Gernand et al., 2003; Zhang et al., 2005) correlates with the position of the pericentromere during mitosis and meiosis II. Analysis of dicentric chromosomes revealed hyperphosphorylated H3S10 only at the functional centromere (Houben et al., 1999; Han et al., 2006; Fu et al., 2012), indicating that it is an epigenetic mark for active centromeres. In contrast to the monocentrics, holocentric chromosomes of *Luzula* (Gernand et al., 2003; Nagaki et al., 2005) and *Rhynchospora* (Guerra et al., 2006) were labeled along their entire length with anti-H3S10ph antibody. Thus, in both chromosome types the H3S10/S28 phosphorylations occur where sister chromatids cohere until the onset of anaphase; in holocentric chromosomes the cohesion occurs along the entire chromatids while in monocentrics only at the primary constriction. Pericentromeres of metaphase chromosomes in plants have been shown to be hyperphosphorylated at H3T3 although H3T3ph was also detected as punctuated signals along the entire length of chromosome arms (Caperta et al., 2008). Compared to H3S10ph, which occurred specifically in the primary constriction, H3T3 hyperphosphorylation was observed in the regions at both sides of the primary constriction. Yet another phosphorylation, located at T120 of H2A, has recently been reported as a new marker for centromeric chromatin in plants (Dong and Han, 2012; Demidov et al., 2014). Chromatin phosphorylated at H2AT120 was found to be intermingled with CenH3 containing nucleosomes, resulting in their colocalization on mitotic chromosomes of all investigated species regardless of their chromosome morphology. Thus, the distinct phosphorylation patterns described above are the most contrasting epigenetic marks distinguishing (peri)centromeric chromatin from parts of the chromosome that are not directly interacting with the spindle. Additional differences were revealed via the investigation of the distribution of histone methylations discriminating different types of chromatin. Histone H3K4me1/2/3, which is typically associated with transcriptionally active chromatin, was found depleted, whereas the marks of transcriptionally silent chromatin like H3K9me2 and H3K27me2 were enriched in (peri)centromeric regions of monocentric chromosomes in *A. thaliana* and some other plant species with small genomes (Fuchs et al., 2006, 2008; Fuchs and Schubert, 2012). However, the contrast in H3K4me1/2/3, H3K9me2, and H3K27me2 density between (peri)centromeres and chromosome arms is not evident in plant species with large genomes which display relatively homogeneous distribution of these marks along chromosomes which is disrupted at some loci on chromosome arms due to presence of large blocks of eu- and hetero-chromatin (Houben et al., 2003; Fuchs et al., 2006, 2008; Shi and Dawe, 2006; Fuchs and Schubert, 2012). On the other hand, holocentric chromosomes were found to lack large-scale patterns of eu- and hetero-chromatin marks typically observed in monocentrics (Heckmann et al., 2013).

A novel type of centromere organization, consisting of remarkably extended primary constrictions containing multiple CenH3 domains was first discovered in the pea (*Pisum sativum*) and designated as “meta-polycentric” (Neumann et al., 2012). In pea chromosomes, CenH3-containing chromatin forms 3–5 distinct spots which are located on the poleward regions of primary constrictions, all of them binding to the spindle fibers. These spots are separated by CenH3-lacking chromatin and the whole constrictions delimited by two outermost CenH3 domains vary between 69 and 107 Mbp, thus being several folds larger than the length of any known monocentric centromere. The centromeric domains are almost entirely composed of repetitive DNA sequences belonging to 13 distinct families of satellite DNA and one family of centromeric retrotransposons, all of which are unevenly distributed among pea chromosomes (Neumann et al., 2012). Following their discovery in *Pisum*, meta-polycentric chromosomes were also found in its sister genus *Lathyrus*, contrary to the closely related genera of *Vicia* and *Lens* (all members of the same legume tribe *Fabeae*) which possess monocentric chromosomes (Neumann et al., 2015). Interestingly, the occurrence of expanded centromeres correlated with the presence of two copies of CenH3 genes in both *Pisum* and *Lathyrus*, which was also reported in the holocentric species *C. elegans, L. nivea*, and *R. pubera* (Monen et al., 2005; Moraes et al., 2011; Marques et al., 2015). On the other hand, there is a number of monocentric species including *Arabidopsis lyrata, Hordeum bulbosum, H. vulgare, Mimulus* spp., that also possess two CenH3 copies (Kawabe et al., 2006; Sanei et al., 2011; Finseth et al., 2015). Thus, it is yet to be investigated whether there is any causal link between CenH3 gene duplication and the shift in centromere morphology in *Fabeae*.

In this work, we aimed to delve deeper into the epigenetic and structural features of meta-polycentric chromosomes in *Lathyrus sativus* and *Pisum sativum* by combining immunolabeling with high-resolution fluorescence microscopy. We surveyed the distribution of histone phosphorylation marks known to be associated with (peri)centromeric regions (H3T3ph, H3S10ph, H3S28ph, and H2AT120ph) as well as histone methylations used to distinguish between different chromatin states (H3K4me3, H3K9me2 and H3K27me2). Since centromere organization in meta-polycentric chromosomes is more complex in comparison to monocentric chromosomes, some established terms should be clarified to avoid any misunderstanding. The term centromere is used exclusively for sites which are marked by the presence of kinetochore proteins such as CenH3. The rest of the chromatin in primary constriction is referred to as pericentromere. In cases where centromere and pericentromere cannot be distinguished we used the term (peri)centromere.

## Materials and methods

### Plant material

Seeds of *Pisum sativum* cv. Terno, *Lathyrus sativus* and *Vicia faba* cv. Merkur were obtained from Selgen (Stupice, Czech Republic), Fratelli Ingegnoli (Milano, Italy) and Osiva (Boršov nad Vltavou, Czech Republic), respectively. Seeds of *Arabidopsis thaliana* (ecotype Columbia) were obtained from the European Arabidopsis Stock Centre (Loughborough, UK).

### Chromosome preparation and immunodetection

Chromosomes were isolated from root tip meristem cells synchronized using 1.25 mM hydroxyurea and blocked at metaphase using 15 μM oryzalin as described previously (Neumann et al., 2002, 2015). Suspension of isolated chromosomes was spun on slides using a Hettich centrifuge equipped with cytospin chambers. Meristems intended for squash preparations were also synchronized using hydroxyurea but were not blocked at metaphase. The squash preparations were made in LB01 lysis buffer (15 mM Tris, 2 mM Na_2_EDTA, 80 mM KCl, 20 mM NaCl, 0.5 mM spermine, 15 mM mercaptoethanol, 0.1% Triton X-100, pH 7.5) by squashing root tip meristems fixed in 4% formaldehyde for 25 min at 20°C and digested with 2% cellulase and 2% pectinase in 1 × PBS for 65 min at 28°C. After squashing the meristems and coverslip removal, the slides were washed 5 min in 1 × PBS, 25 min in PBS-Triton buffer (1 × PBS, 0.5% Triton X-100, pH 7.4) and finally 2 × 5 min in 1 × PBS. Nuclei of *A. thaliana* were isolated from young rosette leaves using the protocol for chromosome isolation described above. The plants were cultivated *in vitro* (12 h photoperiod, 22/20°C) on liquid Murashige and Skoog medium (Duchefa) supplemented with 3% sucrose. The same procedures were used for immunolabeling chromosome and nuclei preparations.

Histone dephosphorylation was carried out with λ-phosphatase (New England Biolabs) for 1 h at 30°C in 1 × NEB buffer for protein metallophosphatases supplemented with 1 mM MnCl_2_. The concentration of λ-phosphatase was 10 U/μl. Prior to phosphatase treatment the slides were washed in 50 mM Tris-HCl supplemented with 10 mM NaCl. At the end of incubation, slides were washed 2 × 5 min in 1 × PBS.

Prior to incubation with primary antibody, the slides were incubated in PBS-Tween buffer (1 × PBS, 0.1% Tween 20, pH 7.4) for 25 min at room temperature (RT). The slides were then incubated with primary antibodies diluted in PBS-Tween overnight at 4°C. Following two washes in 1 × PBS for 5 min, the antibodies were detected using the appropriate secondary antibody in PBS-Tween buffer for 1 h at RT. After two washes in 1 × PBS for 5 min and one wash in 1 × PBS-Tween for 5 min, the slides were counterstained with 4′,6-diamidino-2-phenylindole (DAPI) and mounted in Vectashield mounting medium (Vector Laboratories, Burlingame, CA). A list of all antibodies used in this study and their dilution ratios are shown in the Supplementary Table 1.

### Microscopy

For conventional wide-field fluorescence microscopy, we used either a Nikon Eclipse 600 microscope equipped with a DS-Qi1Mc cooled camera or a Zeiss AxioImager.Z2 microscope with an Axiocam 506 mono camera. Images were generated using the NIS Elements 3.0 software program (Laboratory Imaging, Praha, Czech Republic) or ZEN pro 2012 (Carl Zeiss GmbH). Depending on the material used, we inspected at least 100 mitoses, nuclei, or isolated chromosomes per experiment. To analyse the substructure of chromatin beyond the classical Abbe/Raleigh limit (super-resolution), spatial Structured Illumination Microscopy (3D-SIM) was applied using a C-Apo 63 × /1.2 W Korr objective of an Elyra PS.1 microscope system and the software ZEN 2012 black (Carl Zeiss GmbH). Images were captured using the 405, 488, and 561 nm laser lines for excitation and the appropriate emission filters. The degree of co-localization between CenH3-1 and -2 was measured in SIM image stacks and calculated using the Imaris 8.0 (Bitplane) software. SIM image stacks were also used to produce 3D movies with the Imaris 8.0 (Bitplane) and ZEN 2012 black softwares.

## Results

### Histone phosphorylation patterns demarcate distinct zones on meta-polycentric chromosomes

**Figure 1:**
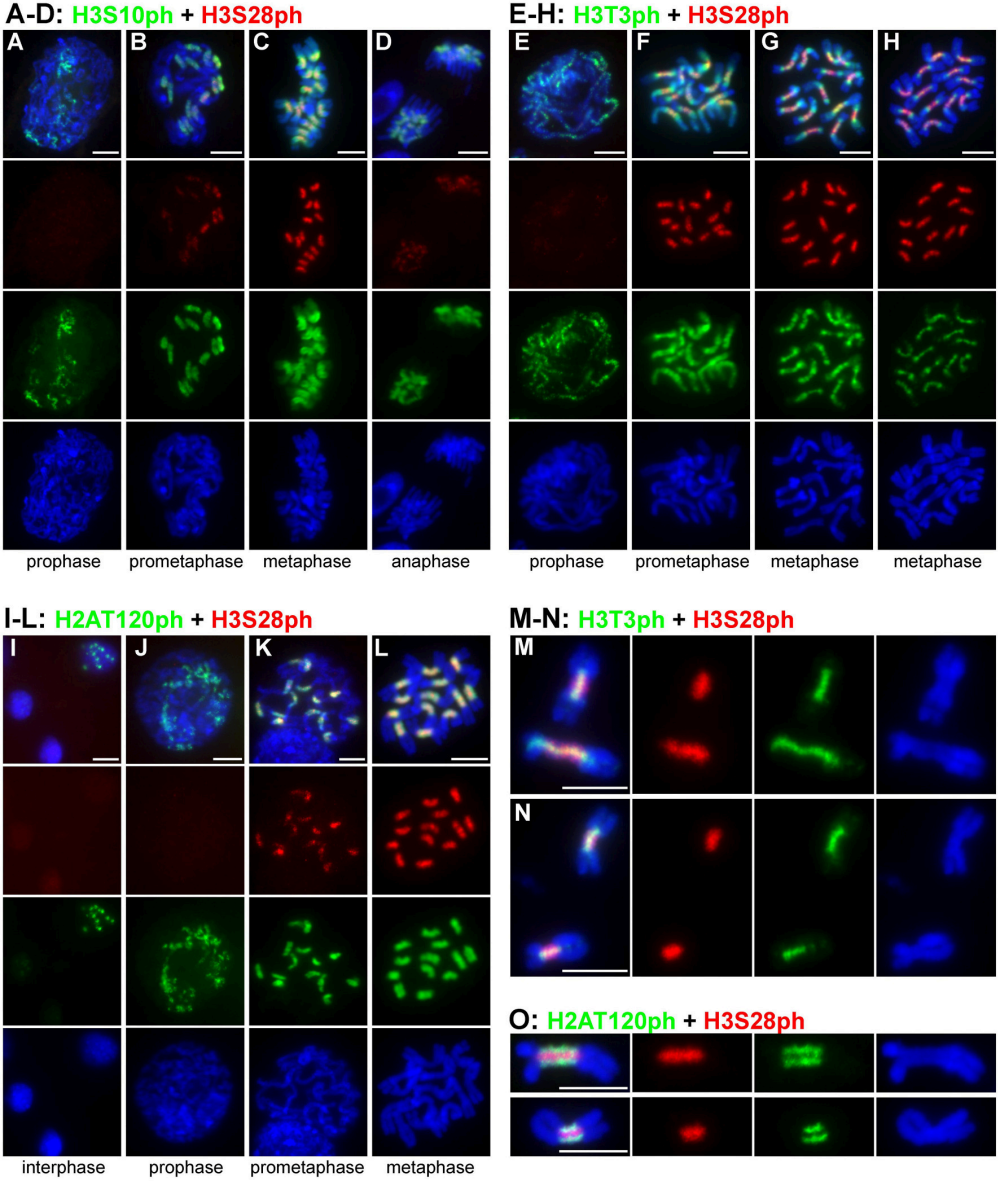
**Distribution of histone phosphorylation marks in *L. sativus* and *P. sativum* analyzed by wide-field fluorescence microscopy**. **(A–D)** H3S10ph and H3S28ph in *L. sativus*. **(E–H)** H3T3ph and H3S28ph in *L. sativus*. **(I–L)** H2AT120ph and H3S28ph in *L. sativus*. **(M–O)** Detection of phosphorylation marks on chromosomes isolated from synchronized meristem cells that were blocked at metaphase using oryzalin. **(M,N)** H3T3ph and H3S28ph on metaphase chromosomes of *L. sativus* **(M)** and *P. sativum* **(N)**. **(O)** H2AT120ph and H3S28ph on metaphase chromosomes of *L. sativus* (upper panel) and *P. sativum* (bottom panel). The images were obtained using wide-field fluorescence microscopy. The chromosomes were stained with DAPI (blue). Bars = 10 μm.

Histone phosphorylation patterns were investigated in parallel in *Lathyrus sativus* and *Pisum sativum* and were found to display similar distributions on chromosomes of both species. With the exception of H2AT120ph, the phosphorylations were not detectable in interphase nuclei and appeared only during individual stages of mitosis. Phosphorylation of histone H3 at serine 10 appeared at early prophase and became most intense by prometaphase. At these two stages of mitosis, H3S10ph presumably occurred in (peri)centromeric regions, as judged from the observation of 14 spatially limited signals in individual prophase cells (Figures 1A,B). On metaphase chromosomes, additional weak signals of H3S10ph were also observed on chromosome arms (Figure 1C). The signals within primary constrictions weakened and became diffuse, remaining stronger at the periphery compared to the inner region. This pattern was also observed during anaphase although the signal intensity was considerably weaker compared to metaphase (Figure 1D).

Histone H3 phosphorylation at threonine 3 was first detected during prophase as weak punctuated signals dispersed along the entire chromosome (Figure 1E). At prometaphase, the signals of H3T3ph were very strong, covering the entire chromosome (Figure 1F). With the progression of mitosis toward metaphase, the signals weakened on the arms of the chromosome but remained intense within and around the pericentromeric region (Figure 1G). On metaphase chromosomes, H3T3ph was limited to the primary constriction and short proximal regions and occurred mainly at the interface of sister chromatids (Figure 1H). The part of the chromosome which displayed the H3T3ph signal corresponded to the region of sister chromatid cohesion (Figures 1M,N).

Compared to H3T3ph and H3S10ph, the histone H3 phosphorylation at serine 28 generally appeared later during the progression of mitosis (Figures 1A,B,E,F,J,K). Although very weak signals of H3S28ph were detected in about 4% (*n* = 53) of prophase chromosomes, they became fully evident in prometaphase. H3S28ph labeling was highly specific for the extended constrictions, reaching its maximum at late prometaphase/metaphase and diminishing by anaphase (Figure 1D).

Whereas, H3T3ph, H3S10ph, and H3S28ph occurred only during mitosis, H2AT120ph was additionally detected in 10% of nuclei (*n* = 240) as discrete spots that most likely corresponded to (peri)centromeres (Figure 1I). This suggests that phosphorylation of H2AT120 begins before the onset of mitosis. It should be noted, however, that the immunosignals in nuclei were very weak. They remained weak in early prophase but greatly intensified by prometaphase and metaphase (Figures 1J–L). On chromosomes with morphologically distinguishable primary constriction, H2AT120ph occurred mainly at its outermost layer and was either absent or considerably depleted in the region at the interface of sister chromatids (Figures 1L,O).

**Figure 2:**
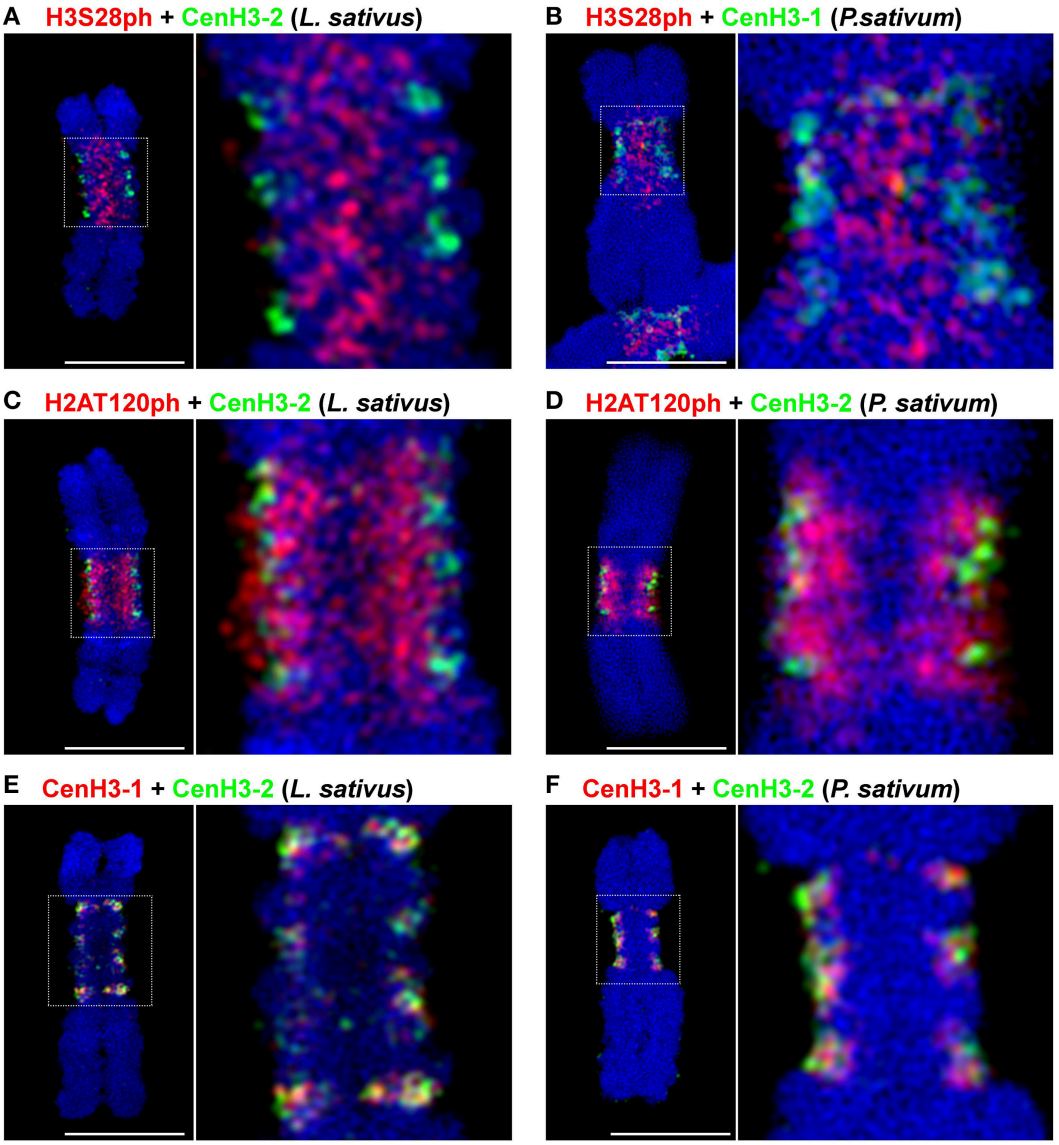
**Simultaneous SIM visualization of epigenetic marks**. **(A)** H3S28ph and CenH3-2 in *L. sativus*. **(B)** H3S28ph and CenH3-1 in *P. sativum* **(C)** H2AT120ph and CenH3-2 in *L. sativus*. **(D)** H2AT120ph and CenH3-2 in *P. sativum*. **(E)** CenH3-1 and CenH3-2 in *L. sativus*. **(F)** CenH3-1 and CenH3-2 in *P. sativum*. For the spatial distribution of the signals on the chromosomes shown in **(D–F)** see Supplementary Movies 1–3. Bars = 5 μm.

**Figure 3:**
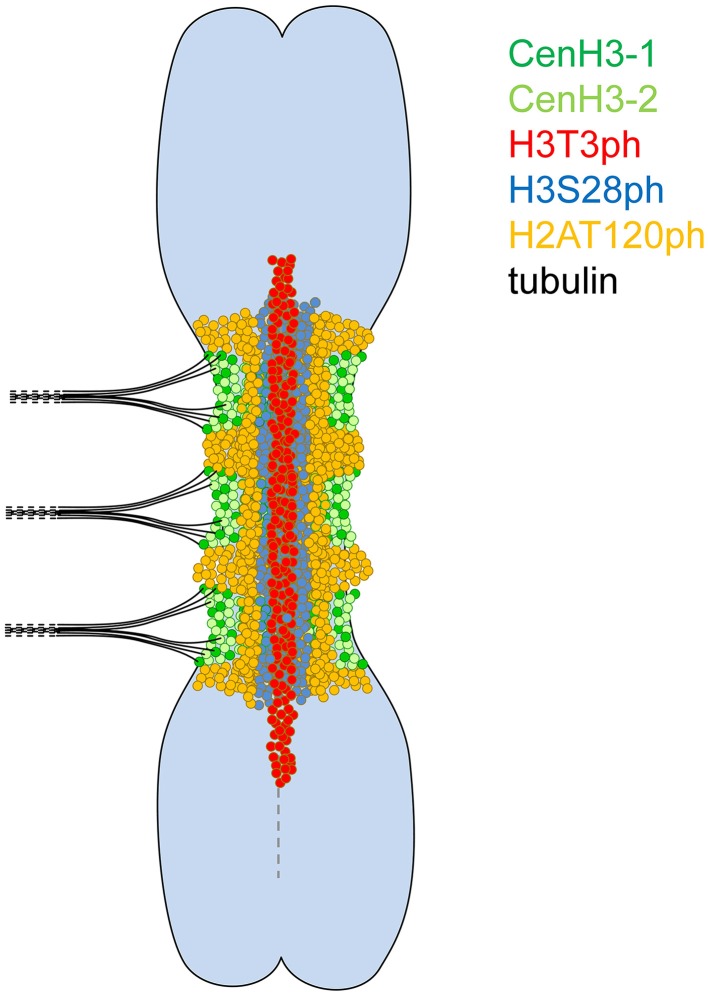
**Model of a metaphase meta-polycentric chromosome**. H3T3ph, H3S28ph, and H2AT120ph occur in contiguous, partially overlapped, longitudinal zones along the entire extended primary constriction. H3T3ph is localized around the interface of sister chromatids within the pericentromere and short proximal segments on both chromosome arms. H3S28ph is widespread throughout the primary constriction, being absent only at the outermost layer. H2AT120ph is located at chromosome periphery near the CenH3-containing domains. In contrast, CenH3 variants are intermingled and organized in multiple, mostly separated domains along the surface of the primary constriction.

Signals of all four tested phophorylation marks were always contiguous on metaphase chromosomes, contrasting with the distribution of CenH3 which often appeared in distinct spots (Neumann et al., 2012, 2015). To investigate mutual localization of histone phosphorylation marks in better detail, we used purified chromosome suspensions which provided higher resolution in immunostaining experiments compared to squash preparations. Moreover, these chromosomes were isolated from synchronized meristem cells blocked at metaphase with oryzalin, ensuring that they represented the same stage of mitosis. Simultaneous detection of H3S28ph with either H3T3ph or H2AT120ph clearly showed that these phosphorylation marks define three parallel partially overlapping zones (Figures 1M–O). Whereas, H3T3ph and H2AT120ph occurred at relatively narrow layers at the sister chromatid interface and chromosome periphery, respectively, H3S28ph was essentially widespread throughout the primary constrictions, being depleted only at the outermost layer. Simultaneous detection of CenH3 with either of H3T3ph, H3S28ph, and H2AT120ph revealed that the CenH3-containing domains are located within the H2AT120ph zone and clearly outside of H3T3ph and H3S28ph zones of primary constriction (Supplementary Figure 1). More accurate visualization of signals using SIM confirmed this observation and further demonstrated that CenH3-containing domains are located at the outer edge of H2AT120ph zone (Figures 2A–D, Supplementary Movie 1). These findings are summarized in a model provided in Figure 3.

### Primary constrictions are depleted in H3K4me3 and H3K27me2 but enriched in H3K9me2

Detection of selected histone H3 methylation marks was performed in order to define the distribution of transcriptionally active chromatin (euchromatin), which is typically associated with H3K4me3, and transcriptionally silent (heterochromatic) regions marked by the presence of H3K9me2 and H3K27me2. Specificity of the antibodies used for these experiments was first confirmed on *Arabidopsis thaliana* nuclei (Supplementary Figure 2). As expected, the H3K9me2 and H3K4me3 antibodies displayed mutually exclusive signals which were in agreement with the distribution of these modifications described earlier (Jasencakova et al., 2003; Fuchs et al., 2006; Fuchs and Schubert, 2012). Expected enrichment of H3K27me2 in heterochromatin (Lindroth et al., 2004; Mathieu et al., 2005; Fuchs et al., 2006; Roudier et al., 2011; Fuchs and Schubert, 2012) was observed in about half of the nuclei whereas the other half of nuclei displayed relatively homogenous distribution of this mark. The latter type of H3K27me2 distribution has so far been observed only by Jacob et al. (2010) who suggested that heterochromatin enrichment of H3K27me2 observed in the other studies is likely due to antibody cross-reactivity with H3K27me1. Considering that both H3K27me1 and H3K27me2 are associated with transcriptionally repressed chromatin (Roudier et al., 2011) and that we aimed at marking this particular type of chromatin, potential cross-reactivity of the antibody with H3K27me1 did not represent a major problem for our study.

**Figure 4:**
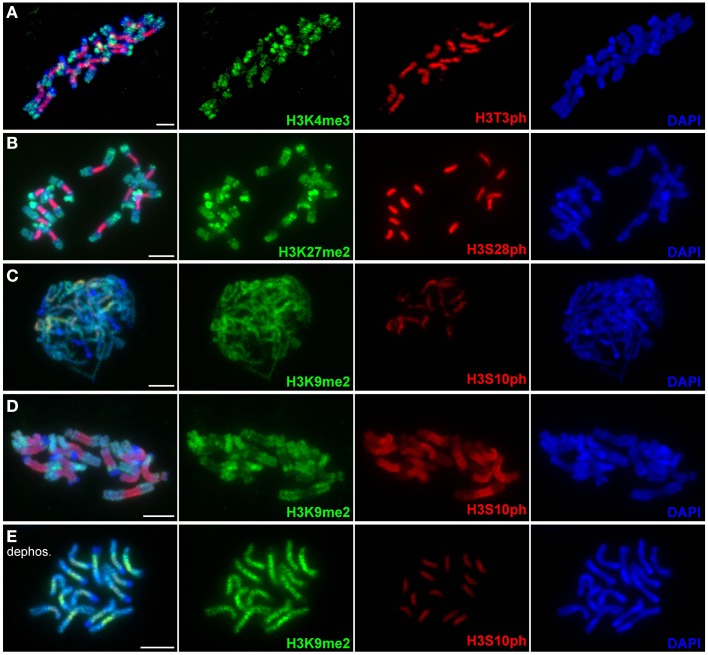
**Distribution of histone methylation marks in *L. sativus* analyzed by wide-field fluorescence microscopy**. Distributions of methylation marks are shown together with phosphorylation marks at the adjacent sites. **(A)** H3K4me3 and H3T3ph labeling on metaphase chromosomes. Note that H3K4me3 signals occur preferentially at the chromosome termini and are depleted in primary constrictions and heterochromatin blocks. **(B)** H3K27me2 (green) and H3S28ph (red) labeling on metaphase chromosomes. The H3K27me2 signals are depleted in primary constrictions and enriched in heterochromatin blocks. **(C)** Detection of H3K9me2 and H3S10ph on prophase chromosomes. Note that signal is depleted exclusively in heterochromatin blocks whereas centromeric regions show equal or even higher signal intensity than chromosome arms. **(D)** Detection of H3K9me2 and H3S10ph on metaphase chromosomes. Note the depletion of H3K9me2 signal in primary constriction. **(E)** Detection of H3K9me2 on metaphase chromosomes upon the treatment with λ-phosphatase. Histone dephosphorylation resulted in stronger H3K9me2 signals in primary constrictions than on chromosome arms. This experiment demonstrated that the depletion of this mark observed on metaphase chromosomes untreated with λ-phosphatase was due to the inability of the antibody to recognize H3K9me2 on histones phosphorylated at adjacent site(s). Bars = 10 μm.

When applied to mitotic chromosomes of *L. sativus* and *P. sativum*, H3K4me3 antibody labeled chromosomes with uneven intensity (Figure 4A and Supplementary Figure 3A). The signals were notably weaker in primary constrictions, whereas the strongest labeling was mostly observed at chromosome termini. H3K27me2 preferentially marked DAPI-positive chromosome bands but occurred at relatively high levels also along the rest of chromosome arms (Figure 4B and Supplementary Figure 3B). Similarly to H3K4me3, the intensity of H3K27me2 labeling was considerably reduced in primary constrictions. Simultaneous detection of H3K27me2 and H3S28ph revealed that the region showing depletion of H3K27me2 was extended further to proximal segments of chromosome arms (Figure 4B). The antibody to H3K9me2 labeled chromosome arms, with the exception of DAPI-positive bands, and only produced weak staining of primary constrictions (Figure 4D). Contrary to this pattern observed on metaphase chromosomes, the (peri)centromeric regions of prophase and early prometaphase chromosomes showed relatively strong H3K9me2 signals (Figure 4C). The apparent depletion of H3K9me2 clearly correlated with increasing phosphorylation at H3S10. Since these histone H3 modifications target adjacent residues, we investigated the possibility that the observed pattern was due to restricted binding of H3K9me2 antibody caused by the presence of S10 phosphorylation [a similar effect was described for other antibodies by Duan et al. (2008) and Jeong et al. (2010)]. In order to remove phosphate groups from histones we pre-treated metaphase chromosomes with λ-phosphatase, a protein phosphatase with activity toward phosphorylated serine, threonine and tyrosine residues. The level of dephosphorylation was tested using antibodies to H3T3ph, H3S10ph, H3S28ph. Compared to the control untreated with λ-phosphatase, the signal intensity of H3T3ph and H3S10ph on chromosomes treated with λ-phosphatase was reduced to 2–5 and 9–11%, respectively, whereas H3S28ph was not detectable at all (Supplementary Figures 4, 5). Detection of H3K9me2 on dephosphorylated chromosomes revealed that the density of H3K9me2 in primary constrictions was actually equal to or up to four-fold higher than on euchromatic regions of chromosome arms (Figure 4E and Supplementary Figure 3C). On the other hand, dephosphorylation had no effect on the weak staining in DAPI-positive bands occurring mainly at subtelomeric regions of some chromosomes of *L. sativus*. This result prompted us to test if the signal patterns of H3K4me3 and H3K27me2 were biased by phosphorylation at adjacent site(s) as well. However, dephosphorylation of the chromosomes with λ-phosphatase had no effect on the pattern of these signals (Supplementary Figure 5). It is not clear, however, whether it was because the binding of these two antibodies is not hampered by phosphorylation at adjacent site(s) or because these two modifications do not occur together.

### The two CenH3 variants are not fully co-localized

In our previous studies, using conventional wide-field fluorescence microscopy we showed that two different CenH3 variants, referred to as CenH3-1 and CenH3-2, are both present in all CenH3 chromatin domains detected on *Lathyrus* and *Pisum* chromosomes (Neumann et al., 2012, 2015). To determine the mutual arrangement of these variants at higher resolution, we analyzed the distribution of these CenH3 variants using specific antibodies and super-resolution microscopy. We found that the variants form distinct, but intermingled subdomains at metaphase chromosomes as well as in interphase nuclei (Figures 2E,F). In *P. sativum*, 54.0% (*n* = 12) of CenH3-1 co-localized to CenH3-2, and conversely 25.8% (*n* = 12) of CenH3-2 co-localized to CenH3-1. In *L. sativus* these values were 41.1% (*n* = 16), and 42.3% (*n* = 16), respectively. However, these relatively high degrees of co-localization did not indicate that CenH3-1 and CenH3-2 are integrated into identical nucleosomes, because even the achieved SIM resolution of 120 nm was not sufficient to provide a definitive answer. To make sure that these results were not influenced by methodological artifacts, we analyzed the signals of each single primary antibody by using two differently labeled secondary antibodies in parallel. Because the signals showed identical shapes and they overlapped completely, such artifacts could be excluded (Supplementary Figure 6). CenH3-containing subdomains were also intermingled with CenH3-lacking chromatin. No preferential arrangement of either of the CenH3 variants toward the surface of primary constriction was observed, suggesting that the two CenH3 variants have identical functions during mitosis (Supplementary Movies 2, 3).

## Discussion

We previously demonstrated that the CenH3-containing domains constitute a very low proportion of chromatin within the extended centromeres of both *P. sativum* and *L. sativus* (Neumann et al., 2015). However, it has remained unknown if the bulk of CenH3-lacking chromatin within the primary constrictions somehow reflects the meta-polycentric organization of CenH3-containing domains. It has been shown in a number of species that histone phosphorylation and methylation marks define different districts surrounding the centromeres of mitotic chromosomes (Wang and Higgins, 2013). Phosphorylation at H3S10 and H2AT120 is assumed to play important roles during mitosis and occurs only around active centromeres of mitotic chromosomes (Funabiki and Wynne, 2013).

In this study, we found that H3T3ph, H3S28ph, and H2AT120ph occurred in contiguous, partially overlapped, longitudinal zones along the primary constrictions of metaphase chromosomes and were ordered from the innermost region at the interface of sister chromatids toward the poleward surface. This contrasts with the organization of CenH3-containing chromatin which forms multiple well separated domains along the surface of primary constrictions. The distribution of H3T3ph, H3S28ph, and H2AT120ph likely reflects differences in the localization of respective kinases, particularly Haspin, Aurora, and Bub1, respectively (Kurihara et al., 2006; Higgins, 2010; Yamagishi et al., 2010; Wang et al., 2012; Wang and Higgins, 2013). In fission yeast and human cells, Haspin is recruited via its interaction with a cohesin complex, which during metaphase resides mainly at centromeres in a region between sister chromatids, whereas loading of Bub1 depends on the interaction with the kinetochore protein KNL1 (Bolanos-Garcia and Blundell, 2011; Marston, 2015). It has been shown in fission yeast, *Xenopus* and human cells that H3T3ph and H2AT120ph mediated by Haspin and Bub1 act in targeting Aurora B to the inner centromere (Kelly et al., 2010; Wang et al., 2010; Yamagishi et al., 2010). In turn, Aurora B phosphorylates Haspin to stimulate phosphorylation of H3T3 and also contributes to kinetochore accumulation of Bub1 (Vigneron et al., 2005; Wang et al., 2011; Funabiki and Wynne, 2013). Importantly, H2AT120ph was shown to serve as a mark for the recruitment of Shugoshin, a protein which protects centromeric cohesin and maintains sister chromatid cohesion at centromeres of metaphase chromosomes (Liu et al., 2015). Taking into account that sister chromatid cohesion and chromosome bi-orientation are coupled (Yamagishi et al., 2010) and that cohesion depends on H2AT120ph, it seems plausible that meta-polycentric chromosomes are stable owing to the contiguous distribution of H2AT120ph along the entire length of the primary constriction. The maintenance of sister chromatid cohesion along the length of the primary constriction of meta-polycentric chromosomes during metaphase is also supported by the contiguous distribution of H3T3ph, which is expected to mirror the distribution of cohesin.

The contiguous distributions of H3S10ph and H3S28ph along the length of extended centromeres of *L. sativus* and *P. sativum* resemble their patterns noted on holocentric chromosomes (Gernand et al., 2003). This observation supports the view that these extended centromeres are functionally similar to holocentric chromosomes with respect to their mechanisms facilitating chromosome division. A notable difference which distinguishes meta-polycentric chromosomes from both holocentric and monocentric chromosomes is the mutual arrangement of H2T120ph and CenH3-containing domains. In *L. sativus* and *P. sativum*, CenH3-containing domains are located at the very periphery of sister chromatids whereas H2AT120ph occurs more toward the inner domain of the primary constriction. In contrast, in mono- and holocentric species, CenH3 and H2AT120ph-specific signals form distinct, partly intermingling structures (Demidov et al., 2014). Another discrepancy between meta-polycentric chromosomes and plant monocentric chromosomes was found in the distribution of H3T3ph on metaphase chromosomes. Whereas, H3T3ph was localized within pericentromeres of metaphase chromosomes in both *P. sativum* and *L. sativus*, in other plant species it was enriched but not restricted to pericentromeres (Houben et al., 2007; Caperta et al., 2008; Ashtiyani et al., 2011). In *L. sativus* and *P. sativum*, the pericentromere-specific signals were observed especially on chromosomes which were isolated from meristem cells blocked at metaphase with oryzalin, thus representing the same stage of mitosis. At earlier stages of mitosis, however, the distribution of H3T3ph was similar to that observed in other plant species.

In addition to the specific occurrence of histone phosphorylation marks, the (peri)centromeres in both *L. sativus* and *P. sativum* differ from the rest of chromosomes at certain histone methylation marks. The combinatorial pattern of H3K4me3, H3K9me2, and H3K27me2 was distinct from both chromatin types generally distinguished on chromosome arms, euchromatin, and heterochromatin (the latter appearing as DAPI-positive bands or dots). In contrast to the heterochromatin blocks which were observed on some chromosome arms of *L. sativus* the (peri)centromeres showed a reduced level of H3K27me2 and a higher level of H3K9me2. On the other hand, compared to euchromatin, the (peri)centromeric regions displayed reduced levels of H3K4me3 and H3K27me2. The low level of H3K4me3 and high level of H3K9me2, marking transriptionally active and silent chromatin, respectively, are consistent with the notion that (peri)centromeres are often gene-poor regions. H3K9 methylation was demonstrated to be required for proper chromosome segregation in fission yeast, mouse, and humans (Bannister et al., 2001; Bernard et al., 2001; McManus et al., 2006; Slee et al., 2012). In our study, histone dephosphorylation experiments demonstrated that a majority of H3K9me2 became phosphorylated at adjacent site(s) during prometaphase/metaphase. It has been proposed that phosphorylation of histone H3 at S10 by AtAurora1 in *A. thaliana* occurs on histones which are pre-methylated at K9 (Demidov et al., 2009). This is known as the “phospho/methyl switch” which seems to play an important role during mitosis in at least some non-plant species (Sawicka and Seiser, 2012). Whereas, phosphorylation of H3S10 is essential for proper segregation of chromosomes during mitosis in *A. thaliana* (Demidov et al., 2009) it is unclear if the presence of H3K9me2 in the (peri)centromere is required for correct mitotic division in plants as well.

Most diploid species studied so far possess only a single CenH3 gene. Besides *Pisum* and *Lathyrus* spp., the presence of two CenH3 genes has been documented only in *Arabidopsis lyrata, Hordeum bulbosum, H. vulgare, Mimulus* spp., *Rhynchospora pubera, Luzula nivea*, and *Caenorhabditis elegans* (Monen et al., 2005; Kawabe et al., 2006; Moraes et al., 2011; Sanei et al., 2011; Finseth et al., 2015; Marques et al., 2015). However, mutual localization of two CenH3 variants on metaphase chromosomes has been investigated at high-level resolution only in *H. vulgare* (Ishii et al., 2015). Similarly to *P. sativum* and *L. sativus*, the two CenH3 variants in *H. vulgare* are organized in distinct but intermingled subdomains. This organization implies that different CenH3 variants do not occur in the same nucleosomes and that nucleosomes possessing the same variant of CenH3 aggregate to form subdomains within centromeres of metaphase chromosomes. Although there is still some controversy regarding the structure of a centromeric nucleosome, most pieces of evidence suggest that the prevalent form is a histone octamer containing two each of histones H2A, H2B, H4, and CenH3 (Tachiwana et al., 2011; Sekulic and Black, 2012; Dunleavy et al., 2013; Hasson et al., 2013). Assuming that the octameric nucleosomes also exist in *P. sativum, L. sativus*, and barley, there must be a mechanism which prevents or impedes loading of different CenH3 variants into the same nucleosome. Crystal structures of (CenH3-H4)_2_ heterotetramer (Sekulic et al., 2010) and complete octameric nucleosome (Tachiwana et al., 2011) revealed that CenH3 forms contacts with both H4 and the other CenH3 molecule (referred to as CenH3′). In human CenH3 (CENP-A) nucleosomes, the CenH3-CenH3′ interface occurs in a region comprising a carboxy-terminal portion of α2 helix, L2 loop and its α3 helix (Tachiwana et al., 2011). It has been shown in flies and humans that mutations disrupting the CenH3-CenH3′ interface eliminate centromere targeting (Sekulic et al., 2010; Zhou et al., 2011; Bassett et al., 2012; Zhang et al., 2012). These observations suggest that the CenH3-CenH3′ interaction is crucial for the assembly and stability of CenH3 nucleosomes and their proper localization. Taking into account that some amino acids in the region comprising the carboxy-terminal portion of α2 helix, L2 loop, and its α3 helix differ between CenH3-1 and CenH3-2 histones in both *P. sativum* and *L. sativus* (Neumann et al., 2012, 2015), it can be speculated that the sequence divergence of the two CenH3 variants may have resulted in their mutual incompatibility. Although an explanation for the spatial separation of CenH3-1 and -2 requires further research, our results demonstrate that divergence of CenH3 histone variants impacts their localization in centromeric chromatin.

## Author contributions

PN, AH, and JM conceived the study and designed the experiments. PN, VS, IF, and JEM performed the experiments. PN and VS analyzed the data. PN, JM, and AH wrote the manuscript with input from VS, IF, and JEM. All authors read and approved the final manuscript.

## Funding

This research was financially supported by grants from the Czech Science Foundation (GAP501/11/1843 to PN), the Czech Academy of Sciences (RVO:60077344) and Deutsche Forschungsgemeinschaft (SPP1384).

### Conflict of interest statement

The authors declare that the research was conducted in the absence of any commercial or financial relationships that could be construed as a potential conflict of interest.
