# Supplementary material for: Epigenetic Histone Marks of Extended Meta-Polycentric Centromeres of Lathyrus and Pisum Chromosomes
Source: Front Plant Sci. 2016 Mar 1;7:234. doi: 10.3389/fpls.2016.00234 (PMC4771749; doi:10.3389/fpls.2016.00234)
Supplement: Supplementary file 4 [file DataSheet1.DOC]

Supplementary Material

**Extended centromeres of *Lathyrus* and *Pisum* chromosomes carry epigenetic marks similar to holocentrics**

**Pavel Neumann, Veit Schubert, Iva Fuková, Jasper E. Manning, Andreas Houben, Jiří Macas^*^**

*** Correspondence:** Jiří Macas, macas@umbr.cas.cz

# Supplementary Data

**Supplementary movie 1. 3D organization of CenH3-2 and H2AT120ph on metaphase chromosome of *P. sativum*.** 2D image of this chromosome is shown in the Figure 2D. CenH3-2 and H2AT120ph are shown in green and red, respectively.

**Supplementary movie 2. 3D organization of CenH3-1 and CenH3-2 on metaphase chromosome of *L. sativus*.** 2D image of this chromosome is shown in the Figure 2E. CenH3-1 and CenH3-2 are shown in red and green, respectively.

**Supplementary movie 3. 3D organization of CenH3-1 and CenH3-2 on metaphase chromosome of *P. sativum*.** 2D image of this chromosome is shown in the Figure 2F. CenH3-1 and CenH3-2 are shown in red and green, respectively.

# Supplementary Figures and Tables

## Supplementary Figures


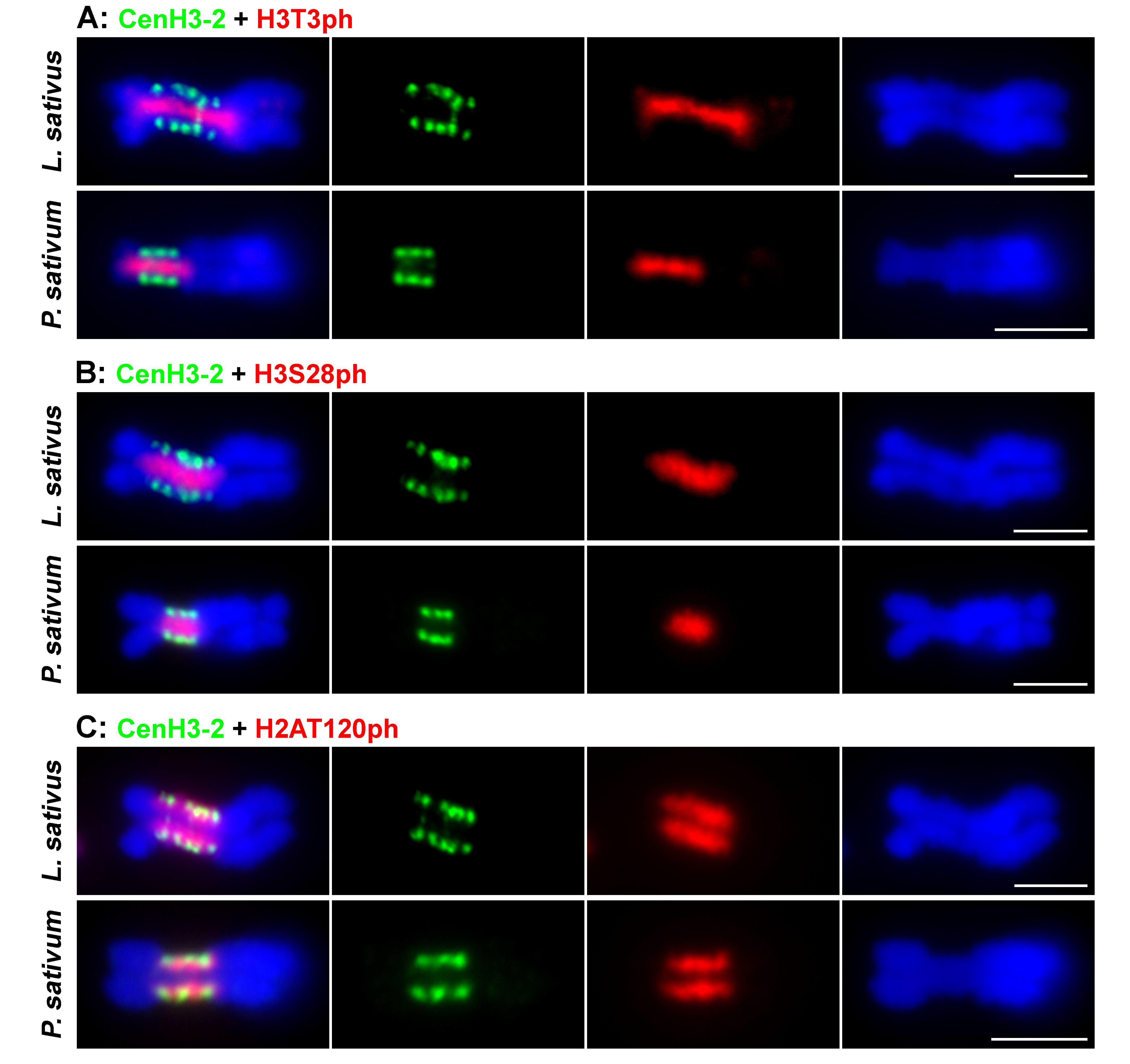
**Figure S1. Simultaneous visualization of histone phosphorylation marks and CenH3-2 in *L. sativus* analyzed by wide-field fluorescence microscopy.** Chromosomes were stained with DAPI (blue). Bars = 5 μm.


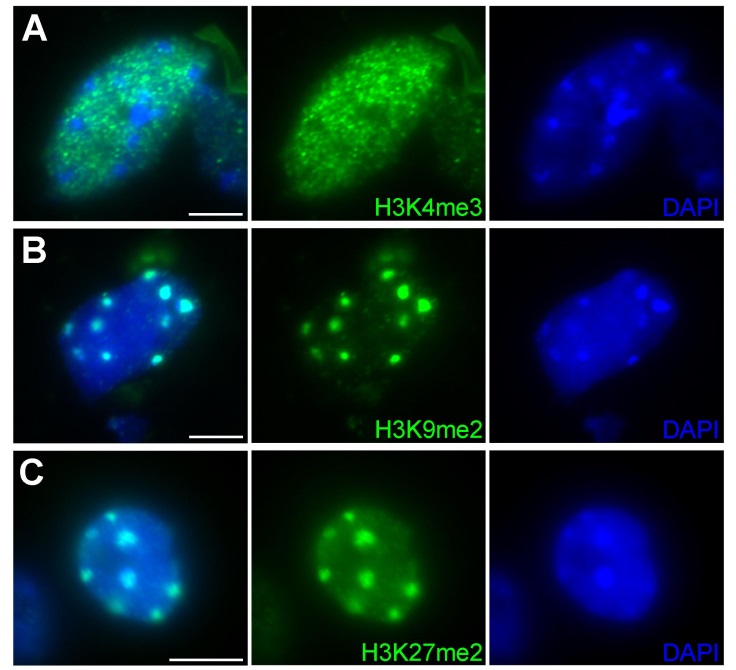


**Figure S2. Detection of histone H3 methylation marks in nuclei isolated from leaves of *A. thaliana*. (A)** H3K4me3. **(B)** H3K9me2. **(C)** H3K27me2. Chromosomes were stained with DAPI (blue). Bars = 5 μm.


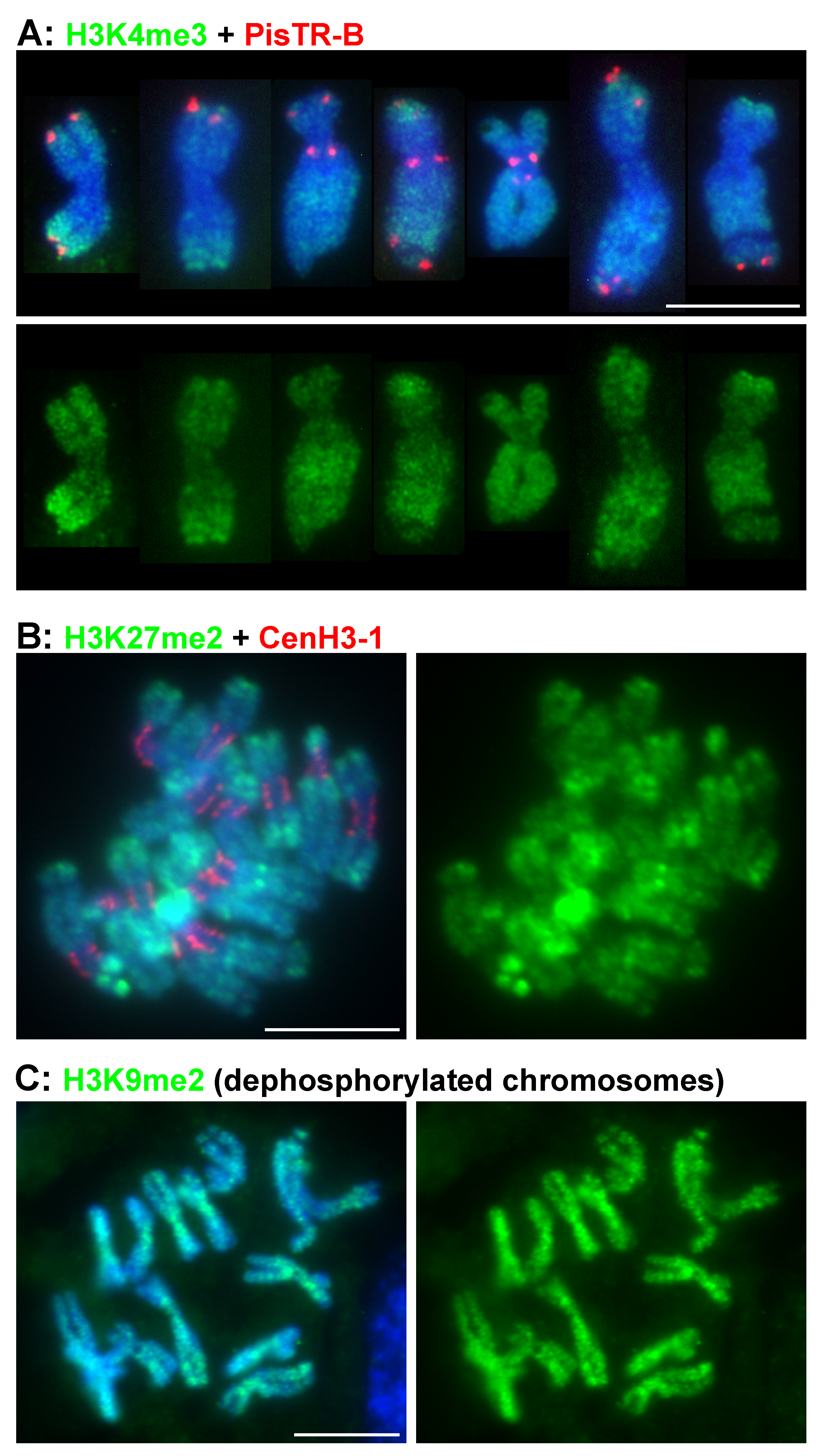


**Figure S3. Distribution of histone H3 methylation marks on chromosomes in *Pisum sativum*. (A)** Detection of H3K4me3 (green) on isolated chromosomes. FISH with PisTR-B probe (red) was carried out following immuno-detection in order to distinguish individual types of pea chromosomes. **(B)** Detection of H3K27me2 (green) on metaphase chromosomes. Centromeres are labeled with CenH3-1 (red). **(C)** Detection of H3K9me2 on metaphase chromosomes pretreated with λ-phosphatase. Chromosomes were stained with DAPI (blue). Bars = 10 μm.


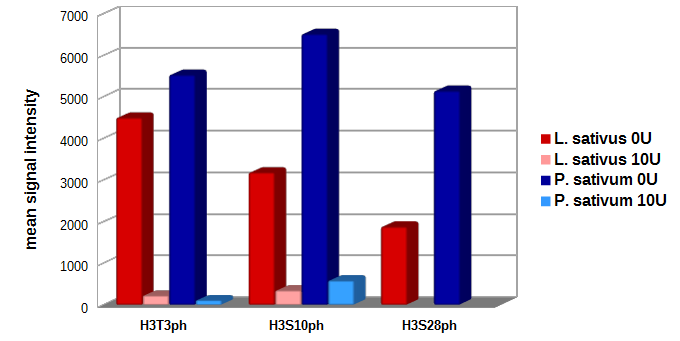
**Figure S4. Dephosphorylation test.** The effect of λ-phosphatase treatment on dephosphorylation of histone H3 was determined for H3T3ph, H3S10ph and H3S28ph. Metaphase chromosomes of *L. sativus* and *P. sativum* were treated with λ-phosphatase at the concentration of 10 U/μl or 0 U/μl (untreated controls). Signals of each phosphorylation mark on chromosomes treated and untreated with λ-phosphatase were captured using the same setup of the microscope to allow for mutual comparison of their intensities. A total of 45 chromosomes were analyzed in each variant.


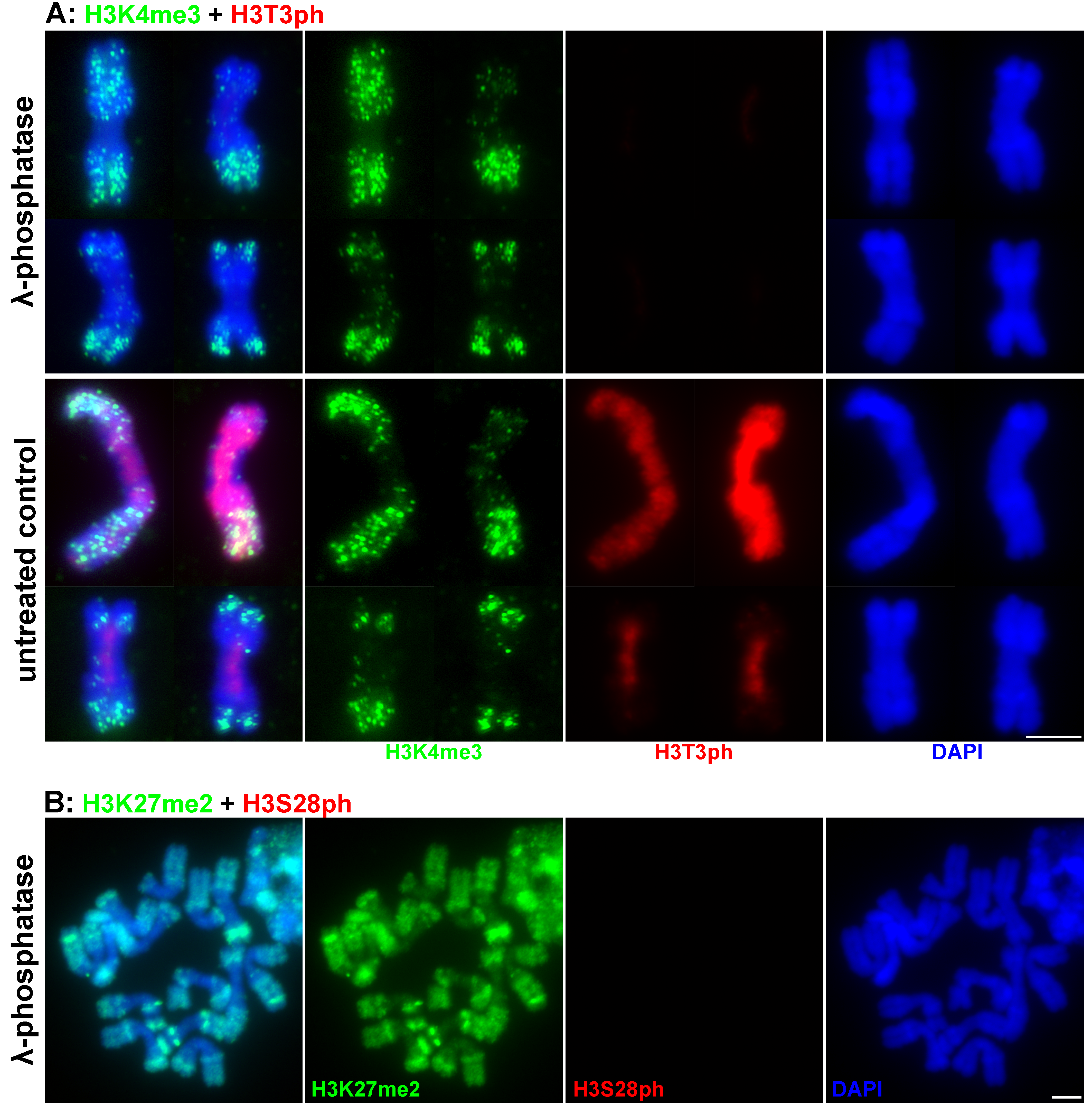
**Figure S5. Detection of H3K4me3 and H3K27me2 on chromosomes of *L. sativus* treated with λ-phosphatase.** **(A)** Examples of H3K4me3 distribution on metaphase chromosomes treated with λ-phosphatase at the concentration of 10 U/μl or 0 U/μl (untreated control). H3T3ph was detected to assess the degree of dephosphorylation. All signals were captured using the same setup of the microscope to allow for mutual comparison of their intensities. The seeming absence of H3T3ph on chromosomes treated with λ-phosphatase is due to low intensity of signals which were not adjusted to show contrast to the untreated control. Note that the patterns of H3K4me3 signals were not affected by chromosome dephosphorylation. **(B)** Distribution of H3K27me2 on metaphase chromosomes treated with λ-phosphatase. Complete dephosphorylation of histone H3 at S28 had no effect on the pattern of H3K27me2 signals which remained depleted in primary constrictions and enriched in heterochromatin blocks (for comparison, see Figure 4B). Bars = 5 μm.


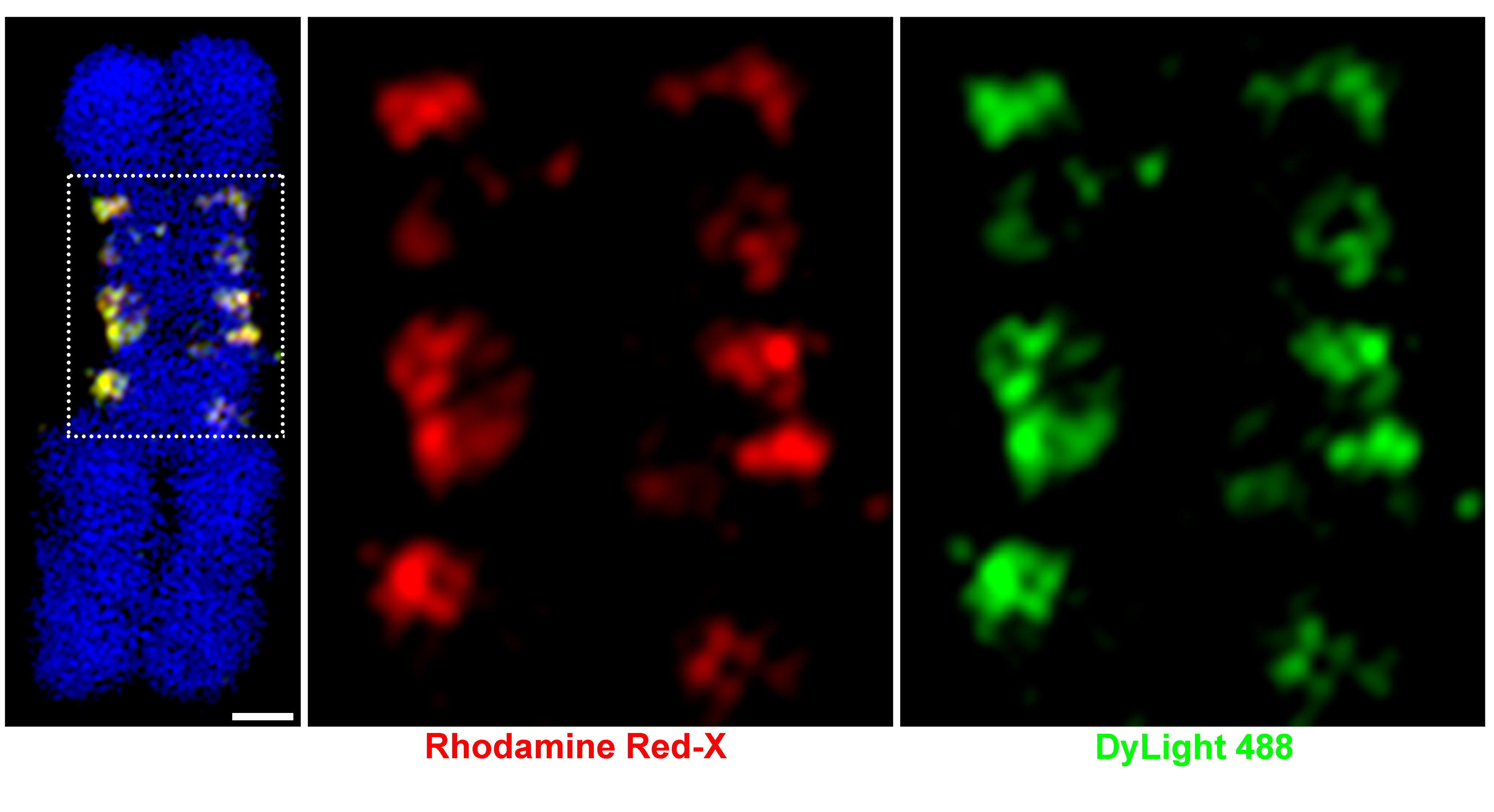
**Figure S6. SIM visualization of CenH3-2 signals detected using two differently labeled secondary antibodies.** Note that the signals fully overlap. Chromosomes were stained with DAPI (blue). Bar = 1 μm.

## Supplementary Tables

## Supplementary Table 1. List of primary and secondary antibodies.

| **Antibody** | **Company** | **Catalog number** | **Dilution** | **Fluorochrome** | **type** | **host** |
| --- | --- | --- | --- | --- | --- | --- |
| CenH3-1^a^ | Genscript | - | 1:5000 | - | primary | rabbit |
| CenH3-2^a^ | Genscript | - | 1:1000 | - | primary | chicken |
| H3K4me3 | Abcam | ab1012 | 1:100 | - | primary | mouse |
| H3K9me2 | Abcam | ab1220 | 1:100 | - | primary | mouse |
| H3K27me2 | Abcam | ab24684 | 1:100 |  | primary | rabbit |
| H3S28ph | Sigma-Aldrich | H9908 | 1:500 | - | primary | rat |
| H2AT121ph | MYBioSource | MBS852710 | 1:1000 | - | primary | rabbit |
| H3T3ph | Merck Millipore | 07-424 | 1:500 | - | primary | rabbit |
| anti-chicken | Jackson ImmunoResearch | 103-485-155 | 1:500 | DyLight 488 | secondary | goat |
| anti-chicken | Jackson ImmunoResearch | 103-295-155 | 1:500 | Rhodamine Red-X | secondary | goat |
| anti-rabbit | Jackson ImmunoResearch | 111-295-144 | 1:500 | Rhodamine Red-X | secondary | goat |
| anti-rabbit | Jackson ImmunoResearch | 111-485-144 | 1:500 | DyLight 488 | secondary | goat |
| anti-mouse | Abcam | ab6785 | 1:100 | Fluorescein isothiocyanate | secondary | goat |
| anti-rat | Jackson ImmunoResearch | 112-295-143 | 1:500 | Rhodamine Red-X | secondary | goat |

^a^ Antibodies to CenH3-1 and 2 were custom-produced as described previously (Neumann et al., 2012).
